# Effects of Fish Oil and Grape Seed Extract Combination on Hepatic Endogenous Antioxidants and Bioactive Lipids in Diet-Induced Early Stages of Insulin Resistance in Rats

**DOI:** 10.3390/md18060318

**Published:** 2020-06-16

**Authors:** Núria Taltavull, Bernat Miralles-Pérez, Maria Rosa Nogués, Sara Ramos-Romero, Lucía Méndez, Isabel Medina, Josep Lluís Torres, Marta Romeu

**Affiliations:** 1 Department of Basic Medical Sciences, Pharmacology Unit, Functional Nutrition, Oxidation, and Cardiovascular Disease (NFOC-SALUT) Group, Universitat Rovira i Virgili, C/Sant Llorenç 21, E-43201 Reus, Spain; nuria.taltavull@urv.cat (N.T.); mariarosa.nogues@urv.cat (M.R.N.); marta.romeu@urv.cat (M.R.); 2 Institute of Advanced Chemistry of Catalonia (IQAC-CSIC), C/ Jordi Girona 18-26, E-08034 Barcelona, Spain; sara.ramos@iqac.csic.es (S.R.-R.); joseplluis.torres@iqac.csic.es (J.L.T.); 3 Department of Cell Biology, Physiology & Immunology, Faculty of Biology, University of Barcelona, E-08028 Barcelona, Spain; 4 Institute of Marine Research (IIM-CSIC), C/ Eduardo Cabello 6, E-36208 Vigo, Spain; luciamendez@iim.csic.es (L.M.); medina@iim.csic.es (I.M.)

**Keywords:** omega-3, proanthocyanidins, polyphenols, antioxidants, diacylglycerol, ceramide

## Abstract

Diacylglycerols (DAG) and ceramides have been suggested as early predictors of insulin resistance. This study was aimed to examine the combined effects of fish oil (FO) and grape seed extract (GSE) on hepatic endogenous antioxidants, DAG and ceramides in diet-induced early stages of insulin resistance. Thirty-five rats were fed one of the following diets: (1) a standard diet (STD group), (2) a high-fat high-sucrose diet (HFHS group), (3) an HFHS diet enriched with FO (FO group), (4) an HFHS diet enriched with GSE (GSE group) or (5) an HFHS diet enriched with FO and GSE (FO + GSE group). In the liver, endogenous antioxidants were measured using spectrophotometric and fluorometric techniques, and non-targeted lipidomics was conducted for the assessment of DAG and ceramides. After 24 weeks, the FO + GSE group showed increased glutathione peroxidase activity, as well as monounsaturated fatty acid and polyunsaturated fatty acid-containing DAG, and long-chain fatty acid-containing ceramides abundances compared to the STD group. The FO and GSE combination induced similar activation of the antioxidant system and bioactive lipid accumulation in the liver than the HFHS diet without supplementation. In addition, the FO and GSE combination increased the abundances of polyunsaturated fatty acid-containing DAG in the liver.

## 1. Introduction

Early stages of insulin resistance are characterized by increased insulin secretion from pancreatic β-cells for maintaining glucose homeostasis under overfeeding conditions [1]. Concretely, insulin resistance is a common feature in metabolic diseases such as obesity, type 2 diabetes and non-alcoholic fatty liver disease (NAFLD), and is related to disturbances in lipid metabolism [2].

Several studies in both rodents and humans support the hypothesis that the aberrant accumulation of bioactive lipids such as diacylglycerol (DAG) and ceramide in the liver is a key step in the pathogenesis and progression of insulin resistance and NAFLD [3]. Indeed, DAG and ceramide species have been suggested as early predictors of metabolic diseases [3,4]. DAG acts as a signal messenger in the cell [5], and ceramide is a signaling lipid and a precursor for molecules involved in the integrity of cell membranes [6]. The synthesis of DAG and ceramide species is stimulated during saturated fatty acid and sucrose overload [7,8,9,10]. At the same time, mitochondrial fatty acid oxidation is promoted to prevent lipid accumulation in the liver under fat overload [11]. This fact might lead to increased reactive oxygen species production and, as a consequence, oxidative damage to lipids, proteins and DNA [11].

Dietary ω-3 polyunsaturated fatty acids (ω-3 PUFA), specifically eicosapentaenoic (EPA, 20:5) and docosahexaenoic (DHA, 22:6) acids, exert antioxidant [12] and anti-inflammatory [13,14] effects on the organism. In addition, dietary supplementation of ω-3 PUFA-rich fish oil (FO) attenuates the development of insulin resistance in rodents under overfeeding conditions [15,16]. However, as the PUFA are highly prone to oxidation leading to irreversible cellular and tissue damage, the addition of antioxidants to PUFA-rich products may be required [17]. Concretely, a grape seed extract (GSE) rich in proanthocyanidins shows high-antioxidant capacity by scavenging free radicals [18]. In this context, the combined administration of PUFA and GSE prevents the oxidation of PUFA during digestion as well as in cell membranes [18,19].

Previously, we described the combined effects of FO and GSE on metabolic health in rats fed a high-fat high-sucrose (HFHS) diet [20,21,22,23]. Concretely, the combination of both ingredients attenuates plasma hyperinsulinemia and hyperleptinemia, decreases liver inflammation and improves blood antioxidant status better than individual supplementations under overfeeding conditions [20,21,22,23]. However, its effects on endogenous antioxidants, DAG and ceramide species in the liver have not been assessed yet.

The aim of this study was to examine the effects of FO and GSE combination on hepatic endogenous antioxidants, DAG and ceramides in diet-induced early stages of insulin resistance in rats.

## 2. Results

### 2.1. Biometric and Biochemical Data

As we previously described [23], the HFHS diet significantly increased the body weight, the perigonadal white adipose tissue weight, the fasting plasma insulin (FI) concentration and the Homeostatic Assessment Model of Insulin Resistance (HOMA-IR) value compared to the standard (STD) diet. Although the FO + GSE group showed increased biometric values compared to the STD group, the FI concentration and the HOMA-IR value were similar between the two groups. On the contrary, individual FO and GSE supplementations did not attenuate either hyperinsulinemia or increased HOMA-IR induced by the HFHS diet compared to the STD diet. Although the HFHS group showed a similar plasma triacylglycerol concentration than the STD group, the individual GSE supplementation increased triacylglycerol in plasma compared all the other groups. The liver weight and the FG concentration showed no significant differences between STD- and HFHS-fed animals (Table S1) [23].

### 2.2. Hepatic Endogenous Antioxidants

All groups fed an HFHS diet, with or without supplementation, presented increased glutathione peroxidase (GPx) activities compared to the STD group. Concretely, the GSE group showed the highest GPx activity. The superoxide dismutase (SOD), catalase (CAT), reduced glutathione (GSH) and oxidized glutathione (GSSG) remained unchanged among the groups by the end of the study (Table 1).

### 2.3. Hepatic Histological Analysis

**Figure 1:**
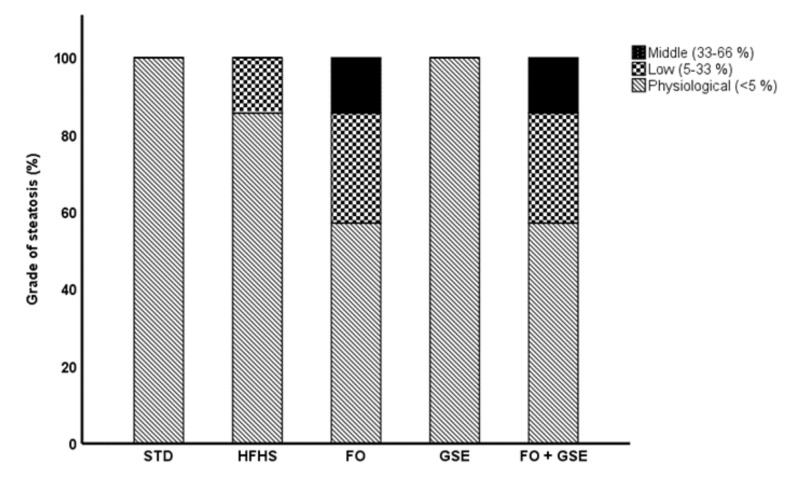
Hepatic steatosis. Results were expressed as frequencies. Abbreviations: STD, Standard; HFHS, High-Fat High-Sucrose; FO, Fish Oil; GSE, Grape Seed Extract. Steatosis was found in no zonal localization in all cases. No significant differences were found among groups.

The liver histological analysis showed that the HFHS diet, with or without supplementation, did not induce either steatosis or pro-inflammatory cell infiltration in the liver by the end of the study. However, FO supplementation, either individual or in combination with GSE, tended to promote fat accumulation compared to the individual GSE supplementation and the STD diet (Figure 1). Steatosis was found in no zonal localization in all cases.

### 2.4. Hepatic Bioactive Lipids

A total of 28 different lipid species were identified in the liver samples: 19 DAGs and nine ceramides (Table S2).

**Figure 2:**
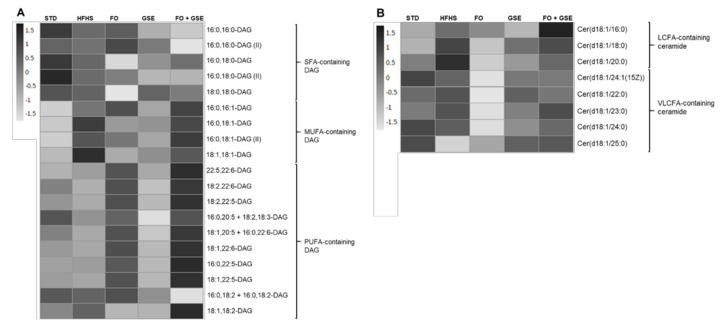
Hepatic bioactive lipids after 24 weeks of dietary intervention. A non-targeted lipidomic approach was carried out on six–seven rats of each group. Results were expressed as heatmaps where rows represent the individual lipid species and columns represent the dietary groups. Significant differences in individual species among groups were commented in the text. (**A**) Diacylglycerol species. (**B**) Ceramide species. Abbreviations: STD, Standard; HFHS, High-Fat High-Sucrose; FO, Fish Oil; GSE, Grape Seed Extract; DAG, Diacylglycerol; SFA, Saturated Fatty Acid; MUFA, Monounsaturated Fatty Acid; PUFA, Polyunsaturated Fatty Acid; Cer, ceramide; LCFA, Long-Chain Fatty Acid; VLCFA, Very Long-Chain Fatty Acid.

Regarding saturated fatty acid (SFA)-containing DAG species, 16:0,16:0-DAG (II) was significantly decreased in the FO + GSE group compared to the other groups. On the contrary, the FO + GSE group as well as GSE, HFHS and STD groups showed a significantly increase of 18:0,18:0—DAG compared to FO. No other significant differences were found in the abundance of the SFA-containing DAG species among the groups (Figure 2A).

Of the monounsaturated FA (MUFA)-containing DAG species detected, 16:0,16:1-DAG was higher in FO + GSE, FO and HFHS than in the STD group. In addition, 16:0,16:1-DAG was increased in the FO + GSE group compared to GSE. The FO + GSE and HFHS groups also increased 16:0,18:1—DAG and 16:0,18:1—DAG(II) compared to the STD group. Likewise, HFHS showed higher 16:0,18:1—DAG and 18:1,18:1—DAG than both the FO and GSE groups. 18:1,18:1—DAG was also increased in the HFHS group compared to the STD group (Figure 2A).

Regarding polyunsaturated FA (PUFA)-containing DAG species, 22:5,22:6—DAG was increased in FO + GSE, FO and GSE compared to STD. Moreover, FO supplementation, either individually or in combination with GSE, increased 22:5,22:6—DAG compared to the GSE and HFHS groups. However, the HFHS group decreased 22:5,22:6—DAG compared to the STD group. FO-supplemented groups showed higher 18:2,22:6—DAG and 18:2,22:5—DAG than the other groups. On the contrary, both GSE and HFHS groups showed lower 18:2,22:6—DAG and 18:2,22:5—DAG than the STD group. Furthermore, 18:2,22:6—DAG was decreased in the GSE group compared to the HFHS group. FO-supplemented groups also presented higher 18:1,20:5 + 16:0 22:6—DAG and 18:1,22:6—DAG than the other groups. In addition, 18:1,20:5 + 16:0 22:6—DAG was decreased in the GSE group compared to the STD group. 18:1,22:5—DAG and 16:0,22:5—DAG was increased in both FO-supplemented groups compared to the other groups. On the contrary, the GSE group showed lower 18:1,22:5—DAG and 16:0,20:5 + 18:2,18:3—DAG than the other groups. No other significant differences were found in abundance of PUFA-containing DAG species among the groups (Figure 2A).

As far as long-chain FA (LCFA)-containing ceramide species are concerned, Cer(18:1/16:0) was significantly increased in FO + GSE and HFHS groups compared to the STD group. Moreover, the FO + GSE group showed significantly higher abundance of Cer(18:1/16:0) than GSE, FO and HFHS groups. The FO and HFHS groups also increased the Cer(18:1/16:0) compared to the GSE group. Cer(18:1/18:0) was higher in the FO + GSE and HFHS groups than in the FO and STD groups (Figure 2B). Cer(18:1/18:1(9Z)) was detected in the liver of the rats fed an HFHS diet, but not in those rats fed an STD diet. No other significant differences were found in the abundance of LCFA-containing ceramide species among the groups.

All groups showed significantly increased very LCFA (VLCFA)-containing ceramides, Cer(d18:1/24:1(15Z)), Cer(d18:1/22:0), Cer(d18:1/23:0) and Cer(d18:1/24:0), compared to the FO group. In addition, the FO + GSE and HFHS groups showed higher Cer(d18:1/23:0) than the GSE group. No other significant differences were found in the abundance of VLCFA-containing ceramide species among the groups (Figure 2B).

## 3. Discussion

We previously described that HFHS diet induces early stages of insulin resistance in the same cohort of rats after 24 weeks, including increased perigonadal adipose tissue content, plasma hyperinsulinemia and plasma hyperleptinemia (Table S1) [23]. The combination of FO and GSE attenuates the metabolic features induced by an HFHS diet (Table S1), decreased liver inflammation, improved blood antioxidant status and increased plasma adiponectin under overfeeding conditions [20,21,22,23].

The present study examined the effects of an FO and GSE combination on hepatic endogenous antioxidants, DAG and ceramides as early biomarkers of insulin resistance. In the liver, HFHS diet induced, first, an early antioxidant response (Table 1) and, second, MUFA-containing DAG and LCFA-containing ceramide accumulation (Figure 2A and 2B) without neither significant steatosis (Figure 1) nor pro-inflammatory cell infiltration. Moreover, HFHS diet decreased several PUFA-containing DAG species compared to STD diet (Figure 2A).

FO + GSE group as well as all the other groups fed an HFHS diet showed moderately higher hepatic antioxidant response than STD (Table 1). This fact could be due to increased mitochondrial β-oxidation in all groups fed an HFHS diet, as previously reported [24]. Antioxidant response is mediated via activation of nuclear factor-erythroid 2-related factor 2 (NrF2) signaling pathway [25] to protect against increased reactive oxygen species production. In addition, both dietary ω-3 PUFA (FO) [26] and proanthocyanidins (GSE) [27] are well-known NrF2 activators. Concretely, the GSE group showed the highest GPx activity in the liver among the groups fed an HFHS diet (Table 1). Consistent with our observations, other authors have showed that the GSE supplementation enhances the expression of antioxidant enzymes in HepG2 cells [28]. Nevertheless, other studies have also suggested that the GSE supplementation reverts the obesity-induced endogenous antioxidant response in Zucker rats by acting as a scavenger of reactive oxygen species [29].

The FO + GSE and FO groups showed a tendency, even though not statistically significant, for lipid accumulation in the liver as assessed by the histological examination compared to the STD group (Figure 1). In accordance with our results, Feillet-Coudray et al. [30] showed no significant differences on hepatic lipid accumulation between fish oil- and lard oil-fed rats. However, other studies have shown that the FO supplementation decreased lipid accumulation in the liver by promoting fatty acid β-oxidation via activation of the peroxisome proliferator-activated receptor α [31,32]. On the other hand, the GSE group showed the lowest lipid accumulation under overfeeding conditions (Figure 1), but showing the highest plasma triacylglycerol concentration among all the groups (Table S1) [23]. In contrast, previous studies have showed that the GSE supplementation decreases the expression of hepatic lipogenic enzymes as well as the triacylglycerol secretion, lowering plasma lipids in Wistar rats fed high-fat diet [33,34].

The non-targeted lipidomic analysis on hepatic DAG and ceramide species showed clearly differentiated hepatic lipid profile between the HFHS and STD groups. As we have mentioned above, the HFHS diet increased the relative abundances of numerous MUFA-containing DAG and decreased several PUFA-containing DAG species compared to the STD diet (Figure 2A). These findings were similar to those of previous studies that had investigated rodents on a high-fat diet [10,35,36]. The hepatic accumulation of DAG species in the HFHS group was likely due to the lipid composition of the HFHS diet, mainly consisting of oleic acid (18:1 n-9), palmitic acid (16:0) and stearic acid (18:0). In contrast, the STD diet was largely made up of linoleic acid (18:2 n-6), which could explain the differences in the hepatic PUFA content between HFHS and STD groups. Additionally, according to Ciapaite et al. [24], adaptive responses (such as the fatty acid desaturation from SFA to MUFA after dietary saturated fat overload) may explain the differences observed in the hepatic MUFA content between HFHS and STD groups. The sucrose overload of HFHS diet might also be responsible for the accumulation of lipids in the liver by de novo synthesis of DAG species [37]. This accumulation of hepatic DAG species in HFHS group could lead to insulin resistance and the development of hepatic steatosis [2] via protein kinase C ε (PKCε) activation and the resulting inhibition of the insulin receptor, as previously described [5,38].

Individual FO and GSE supplementations attenuated the HFHS-diet-induced MUFA-containing DAG accumulation. Even though the combination of both FO and GSE promoted the accumulation of MUFA-containing DAG in the liver. In contrast, the combination of both ingredients markedly decreased the 16:0,16:0-DAG (II), an SFA-containing DAG. FO supplementation, either individually or in combination with GSE, increased PUFA-containing DAG compared to the other three groups (Figure 2A). As we previously described in the same cohort of rats [21], FO supplementation increases the total content of ω-3 PUFA in the liver in accordance with its lipid composition. On the contrary, the individual GSE supplementation significantly decreased several PUFA-containing DAG species in the liver compared to all the other groups (Figure 2A). As we have mentioned above, this fact could be related to the decreased hepatic lipogenesis [33,34] and the increased triacylglycerol secretion (Table S1) [23] induced by GSE.

Moreover, HFHS diet induced hepatic LCFA-containing ceramide accumulation, specifically Cer(d18:1/16:0) and Cer(d18:1/18:0) species, but not modified VLCFA-containing ceramide abundances compared to the STD diet (Figure 2B). These findings are similar to those of previous studies that had investigated rodents on a high-fat diet [10,35,36,39,40]. Nevertheless, other authors have not reported any changes in total hepatic ceramide content after a 3-day fat overload in rats [41] and an 8-week overload in mice [42]. In contrast, Holland et al. found that acute SFA infusions increase the total ceramide abundance in mice liver [43]. Total hepatic ceramide quantification may not reveal associations between individual ceramide species and metabolic diseases [35]. In this respect, recent studies have shown that several ceramide species have distinct roles in the progression of metabolic disorders and that this role depends on their acyl-chain length [35,44]. High ratios of LCFA-containing ceramides to VLCFA-containing ceramides in the liver are associated to the progression of metabolic disorders in rodents [35]. Indeed, a high liver content of Cer(d18:1/22:0) and Cer(d18:1/24:0) might prevent hepatic steatosis under fat overload conditions by reducing peroxisome proliferator-activated receptor γ2 content and, as a consequence, reducing CD36 and FSP27 gene expression [45].

Ceramides are generated by de novo synthesis from palmitoyl-CoA and serine or a salvage pathway from complex sphingolipids [6]. Concretely, HFHS diet-induced Cer(d18:1/16:0) and Cer(d18:1/18:0) accumulation could be, at least in part, due to increased ceramide synthase 6 activity [39]. High abundances of these two LCFA-containing ceramide species have been associated with body weight gain, impaired glucose tolerance or insulin resistance and hepatic steatosis in rodents [36,39,40,46,47]. In humans, increased de novo synthesis and accumulation of ceramides in the liver have been associated with severe obesity, hepatic insulin resistance and non-alcoholic steatohepatitis (NASH) [48,49,50]. Additionally, the activation of the salvage pathway may be positively associated with oxidative stress and inflammation in NASH conditions, suggesting that ceramide precursors contribute to the progression from fatty liver to NASH in humans [49]. These metabolic alterations may largely be due to serine/threonine kinase 1 inhibition [6] and the activation of PKC and CD36 gene expressions, which mediate hepatic fatty acid uptake [51]. In this respect, the promotion of LCFA-containing ceramide degradation by ceramidase improves glucose and lipid metabolism in liver and adipose tissue [51].

Although the individual FO supplementation decreased either LCFA- or VCFA-containing ceramides abundances under overfeeding conditions, the combination of FO and GSE did not attenuate the HFHS diet-induced LCFA-containing ceramide accumulation (Figure 2B). A previous study in humans showed that ω-3 PUFA derived from fatty fish decreased plasma ceramide concentration compared to control [52]. In addition, Dong et al. showed that ω-3 PUFA supplementation at 3:1 EPA:DHA ratio prevented hyperhomocysteinemia-induced ceramide accumulation in C57BL/6J mice [53]. Other authors also showed that FO supplementation decreased ceramide accumulation in adipose tissue under overfeeding conditions [9]. Nevertheless, mice fed a ω-3 PUFA-rich diet or a ω-3 PUFA-poor diet showed no difference either in hepatic or plasma ceramide content [54]. The GSE group also presented lower Cer(d18:1/16:0) and Cer(d18:1/23:0) abundances than HFHS one. In agreement with our results, Seo et al. [55] showed that a supplementation with Chardonnay grape seed flour may decrease de novo synthesis of ceramides.

In addition, Cer(18:1/18:1(9Z)) was only detected in the liver of the rats fed HFHS diet, suggesting that it could be directly derived from the milk fat included in this diet type [56].

The increased abundances of hepatic MUFA- and PUFA-containing DAG as well as LCFA-containing ceramide species observed in FO + GSE group (Figure 2A,B) were not accompanied with insulin resistance (Table S1). The DAG mediated-activation of the PKCε may depend on the DAG localization in the cell [57]. In this way, the accumulation of DAG in lipid droplets rather than plasma membrane prevents PKCε activation and, as a consequence, averting hepatic insulin resistance in CGI-58 ASO-treated mice [57]. In addition, the relationship between ceramide content and insulin resistance remains controversial [3]. Thus, in agreement with other authors [5,41], these results could suggest that ceramide accumulation in the liver is not involved in the development of insulin resistance.

## 4. Materials and Methods

### 4.1. Ethical Statement

All animal procedures respected the European Union guidelines (EU Directive 2010/63/EU) for the care and management of laboratory animals, and the relevant permission was obtained from the CSIC Subcommittee of Bioethical Issues (reference no.CEEA-12-007).

### 4.2. Animals and Diets

Thirty-five female Wistar Kyoto rats (147 ± 9 g body weight), 8–10 weeks old, were purchased from Charles Rivers Laboratories (WKY/NCrl, Wilmington, MA, USA). The rats were kept in an insulated room (two or three rats per Makrolon cage; 425 × 265 × 180 mm) with a constantly regulated temperature (22 ± 2 °C), and controlled humidity (50 ± 10%) in a 12-h artificial light cycle.

The rats were randomized into five groups (seven rats per group), and fed one of the following diets for 24 weeks: (1) an STD diet (STD group; based on Teklad Global 14% Protein Rodent Maintenance Diet [3.1 Kcal/g], Harlan Teklad Inc, IN, USA), (2) an HFHS diet (HFHS group; based on TD.08811 45% Kcal Fat Diet [4.8 Kcal/g], Envigo, IN, USA), (3) an HFHS diet enriched with FO (FO group), (4) an HFHS diet enriched with GSE (GSE group), or (5) an HFHS diet enriched with FO and GSE (FO + GSE group). The full composition of the diets is described in the supplementary materials (Tables S3 and S4). The rats were given free access to food and water (Ribes, Barcelona, Spain) throughout the study. FO and GSE contents were chosen as previously described [20,58].

FO with EPA (C20:5 ω-3) and DHA (C22:6 ω-3) in a balanced 1:1 ratio was obtained by mixing the appropriate quantities of the commercial fish oils AFAMPES 121 EPA (AFAMSA, Vigo, Spain), Omega-3 RX (EnerZona, Milan, Italy) and Oligen liquid DHA 80% (IFIGEN-EQUIP 98, S.L., Barcelona, Spain). FO mixture (24 mL/Kg feed) was added to the pellet of FO and FO + GSE groups. Soybean oil (Clearspring Ltd., London, UK) was added to the preparations of STD, HFHS and GSE groups. The GSE (Fine Grajfnol®, powder 98%) was purchased from JF-Natural Product (Tianjin, China), containing ≥ 95% oligomeric proanthocyanidins (UV), which 60% was procyanidin dimer B2 (HPLC) and ≤ 1.5% Ash. Loss on drying was ≤ 5.0%. GSE (1090 mg/Kg feed) was added to the pellet of GSE and FO + GSE groups.

After 24 weeks, the animals were fasted overnight, anesthetized intraperitoneally with ketamine and xylazine (80 mg/kg and 10 mg/kg body weight, respectively) and sacrificed by exsanguination. Blood samples were taken by cardiac puncture. Subsequently, plasma was obtained by centrifugation at 850 × g for 15 min at 4 °C from the blood samples. Perigonadal white adipose tissue and liver were collected, washed with 154 mM sodium chloride solution, cut and weighted. Then, adipose tissue and liver samples were quickly frozen in liquid nitrogen and stored at −80 °C until processing, except one part of liver that was fixed in 4% formaldehyde solution (v/v) for the histological study.

### 4.3. Biometric and Biochemical Data

Body weight (g) was measured daily throughout the study. Fasting blood glucose concentration (FG) was measured by applying the enzyme electrode method using an Ascensia Elite XL glucometer (Bayer Consumer Care AG, Basel, Switzerland). FI concentration was measured using an ELISA kit (Millipore Corporation, Billiberica, MA, USA) [23]. Subsequently, the HOMA-IR was calculated as: FI (mU/L) × FG (mmol/L) / 22.5 [59]. Plasma triacylglycerol was measured using the corresponding spectrophotometric kit (Spinreact, Barcelona, Spain) in a COBAS MIRA Autoanalyzer (Roche Diagnostics System, Madrid, Spain) [60].

### 4.4. Hepatic Histological Analysis

Formalin-fixed liver samples were dehydrated in alcohol and embedded in paraffin. Serial tissue sections (3 μm thick slices) were obtained using a steel knife mounted in a microtome (Microm HN 355S). Sections were stained with hematoxylin/eosin (Harris Hematoxylin, QCA). Hepatic histological examination was performed after hematoxylin/eosin staining and graded as detailed in (supplementary material Table S5).

### 4.5. Hepatic Endogenous Antioxidants

The liver was homogenized on ice in 200 mM sodium phosphate buffer (pH 6.25) and centrifuged at 129,000× *g* for 1 h at 4 °C. In the liver supernatant, total SOD, CAT and GPx activities were measured using spectrophotometric techniques, and GSH/GSSG contents were measured using fluorometric techniques as previously described [61].

### 4.6. Lipid Extraction from the Liver

The liver was lyophilized and homogenized in a porcelain mortar. The lipids were extracted using a method based on Bligh and Dyer [62]. Briefly, 2 ± 0.1 mg of lyophilized liver was mixed with 375 µL of CHCl_3_/MeOH 1:2 (v/v) and 50 µL of 1 mg/L Cer(d18:1,17:0) and rac-1,2-Dipalmitoylglycerol-d5 as internal standards. After shaking in an ultrasonic bath, the lipid and aqueous phases were broken down by the addition of 125 µL of CHCl_3_ and 125 µL of water. After vortex and centrifugation at room temperature, the organic phase (bottom phase) was carefully separated. Aliquots of 100 µL of the organic phase were diluted 1:10 in MeOH/2-propanol 60:40 (v/v). The sample was subjected to lipidomic analysis at the Centre for Omic Sciences, the joint unit of the Universitat Rovira i Virgili and the EURECAT Technology Centre of Catalonia.

### 4.7. HPLC-qTOF Analysis of DAG

The liver lipid extract was injected into a 1290 Infinity UHPLC system coupled to a 6545 qTOF (Agilent Technologies, Santa Clara, CA, USA). The chromatographic column was C8 BEH 150 × 2.1 mm, 1.7 µm from Waters (Milford, MA, USA.). The mobile phases were 0.1% aqueous HCOOH (A) and 0.1% HCOOH in CH_3_CN/2-propanol 60:40 (v/v) (B). The injection volume was 2 µL. The column flow was set at 0.2 mL/min, and the gradients of the mobile phases were 0–1 min 40% B isocratic, 1–10 min 100% B, 10–19 min 100% B isocratic and 19–20 min 40% B. A post-run of 5 min was applied.

Source conditions were 250 °C and 11 mL/min of drying temperature and gas flow, respectively, a nebulizer pressure of 45 psi, a capillary voltage of 3000 V and a fragmentor voltage of 150 V. Acquisition was set in positive mode, and the *m/z* axis was internally calibrated throughout the run with reference masses 112.9855 *m/z* and 1033.9881 *m/z*. Scan data were recorded in a range between 100 and 1400 *m/z*, at an acquisition rate of 2 spec/s. The collision energy applied for targeted MS/MS analysis was 10, 20 and 40 V, and the product ion spectra were recorded in the range 50 to 800 *m/z*, at a scan range of 5 spec/s.

The compounds were identified using the exact mass of the molecular adducts [M + NH_4_]^+^ and [M + Na]^+^ in the MS analysis and the observed product ions of [M − R_1_COOH + H]^+^, [M − R_2_COOH + H]^+^, [R_1_COO + C_3_H_5_O + H]^+^, [R_2_COO + C_3_H_5_O + H]^+^, [R_1_CO]^+^ and [R_2_CO]^+^ in the targeted MS/MS analysis. The fragmentation rules for this kind of compound were taken into account. The DAG species identified were relatively quantified by comparison of the area under the chromatographic peak ratio corresponding to the [M + NH_4_]^+^ extracted ion chromatogram in the MS analysis [63,64,65].

DAG species were divided into three categories: (1) SFA-containing DAG, (2) MUFA-containing DAG and (3) PUFA-containing DAG (Table S2).

### 4.8. HPLC-QqQ Analysis of Ceramides

The liver lipid extract was injected into a 1290 Infinity UHPLC system was coupled to a 6490 QqQ mass spectrometer (Agilent Technologies, Santa Clara, CA, USA). The same chromatographic conditions were used as described above. Electrospray ionization was performed in positive mode by applying a gas temperature and flow of 150 °C and 11 mL/min, respectively. The nebulizer pressure was set at 20 psi, and the sheath gas temperature and flow were 350 °C and 12 L/min, respectively. Capillary voltage was 3000 V and nozzle voltage was 1000 V.

The acquisition was performed in precursor ion mode by scanning a range of *m/z* from 300 to 700 in the first quadrupole at a scan time of 200 ms, and monitoring the characteristic ceramide product ions of 282 and 264 *m/z* in the second quadrupole. The collision energy was set at 30 V for both transitions. The ceramide species were identified by observing both transitions from the parent ions [M + H]^+^ and [M − HO_2_ + H]^+^. After ceramide specific transitions were identified, the samples were measured again using a Multiple Reaction Monitoring (MRM) method, and ceramides were relatively quantified by comparison of the area under the chromatographic peak ratio for the transitions corresponding to the [M + H]^+^ > 264 *m/z* [66,67].

Ceramide species were divided into two categories: (1) LCFA-containing ceramide, and (2) VLCFA-containing ceramide (Table S2).

While DAG and ceramides were being analyzed by LC-MS, blank runs and quality control standards were injected alternately throughout the run sequence. This showed that there was no carryover among the samples, and that the compounds and/or instrumental drift did not significantly degrade during the sequence analysis.

### 4.9. Statistical Analysis

The statistical analysis was performed using the SPSS 25 statistical package (SPSS, Chicago, IL, USA). Biometric, biochemical and endogenous antioxidants data were expressed as mean ± standard deviation (SD), whereas histological results were expressed in frequencies (%). The Shapiro-Wilk test and Levene’s test were used to test for normality and homoscedasticity of data, respectively. Then, the groups were statistically compared by the one-way analysis of variance (ANOVA) or the non-parametric Kruskal-Wallis test followed by a Scheffé post-hoc test or Mann–Whitney U test, respectively. The contingency tables using χ2 statistics for categorical variables. Relative lipid abundances were log-transformed, auto-scaled and expressed as a heatmap using Metaboanalyst 4.0 free-web based tool [68]. The level of statistical significance was set at p-value < 0.05. Results of individual lipid species were corrected using the false discovery rate controlling procedure for multiple comparisons.

## 5. Conclusions

In conclusion, the FO and GSE combination enhanced antioxidant response and promoted MUFA-containing DAG and LCFA-containing ceramide accumulation in the liver. Additionally, FO and GSE combination increased PUFA-containing DAG mainly due to the FO composition. These findings suggested that the beneficial effects of FO and GSE combination on early stages of insulin resistance were not related to DAG or ceramide species. However, further powered studies are needed to determine the molecular and biochemical mechanisms that regulate DAG and ceramide abundances, including different cellular localizations and tissues, in a biological context.

## Figures and Tables

**Table 1:** Hepatic endogenous antioxidants after 24 weeks of dietary intervention.

	STD n = 7	HFHS n = 7	FO n = 7	GSE n = 7	FO + GSE n = 7	*p*-Value
SOD (U/g)	3678.45 ± 663.32	4465.63 ± 609.73	3136.19 ± 813.26	4190.11 ± 820.02	4010.73 ± 943.69	0.033 *
CAT (nmol/g)	273.80 ± 152.39	408.22 ± 233.11	408.39 ± 119.98	357.27 ± 61.85	382.48 ± 98.63	NS ^†^
GPx (U/g)	47.35 ± 7.11	61.41 ± 3.42 ^a^	64.91 ± 10.33 ^a^	83.47 ± 9.82 ^a,b,c^	65.19 ± 16.74 ^a,d^	<0.001 ^†^
GSH (µmol/g)	0.86 ± 0.65	0.76 ± 0.55	0.95 ± 0.49	1.81 ± 0.34	1.41 ± 0.90	0.016 *
GSSG (µmol/g)	2.44 ± 0.75	2.74 ± 0.62	2.21 ± 0.57	2.00 ± 0.27	2.09 ± 0.54	NS *
GSSG/GSH	5.48 ± 4.57	6.72 ± 6.09	3.71 ± 3.59	1.15 ± 0.31	2.15 ± 1.38	NS ^†^

Results were expressed as mean ± standard deviation. Abbreviations: STD, Standard; HFHS, High-Fat High-Sucrose; FO, Fish Oil; GSE, Grape Seed Extract; SOD, Superoxide Dismutase; CAT, Catalase; GPx, Glutathione Peroxidase; GSH, Reduced Glutathione; GSSG, Oxidized Glutathione. * *p*-value was calculated by a one-way ANOVA followed by a Scheffé post-hoc test. ^†^ *p*-value was calculated by the non-parametric Kruskal-Wallis test followed by or Mann–Whitney U test. ^a^; vs. STD group, ^b^; vs. HFHS group, ^c^; vs. FO group, ^d^; vs GSE group.

## Supplementary Materials

The following are available online at https://www.mdpi.com/1660-3397/18/6/318/s1, Table S1: Biometric and biochemical data in rats after six months of dietary intervention, Table S2: Diacylglycerol and ceramide species identified in the rat liver, Table S3: Composition of diets, Table S4: Fatty acid composition of diets, Table S5: Characterization used for the liver histological study.
