# Supplementary material for: Effects of Fish Oil and Grape Seed Extract Combination on Hepatic Endogenous Antioxidants and Bioactive Lipids in Diet-Induced Early Stages of Insulin Resistance in Rats
Source: Mar Drugs. 2020 Jun 16;18(6):318. doi: 10.3390/md18060318 (PMC7345288; doi:10.3390/md18060318)
Supplement: Supplementary file 1 [file marinedrugs-18-00318-s001.zip › supplementary_material/Taltavull-FishOilAndGrapeSeedExtract-TableS1.pdf]

Supplementary data:

Effects of Fish Oil and Grape Seed Extract Combination on Hepatic Endogenous Antioxidants and Bioactive Lipids in Diet-Induced Early Stages of Insulin Resistance in Rats

Núria Taltavull [1], Bernat Miralles-Pérez [1,*], Maria Rosa Nogués [1], Sara Ramos-Romero [2,3], Lucía Méndez [4], Isabel Medina [4], Josep Lluís Torres [2] and Marta Romeu [1]

1. Universitat Rovira i Virgili, Department of Basic Medical Sciences, Pharmacology Unit, Functional Nutrition, Oxidation, and Cardiovascular Disease (NFOC-SALUT) group, C/ Sant Llorenç 21, E-43201 Reus, Spain; nuria.taltavull@urv.cat (N.T.); mariarosa.nogues@urv.cat (M.R.N.); marta.romeu@urv.cat (M.R.N.)
2. Institute of Advanced Chemistry of Catalonia (IQAC-CSIC), C/ Jordi Girona 18-26, E-08034 Barcelona, Spain; sara.ramos@iqac.csic.es (S.R.-R.); josepllluis.torres@iqac.csic.es (J.L.T.)
3. Department of Cell Biology, Physiology & Immunology, Faculty of Biology, University of Barcelona, E-08028 Barcelona, Spain
4. Institute of Marine Research (IIM-CSIC), C/ Eduardo Cabello 6, E-36208 Vigo, Spain; luciamendez@iim.csic.es (L.M.); medina@iim.csic.es (I.M.)

* Correspondence: bernat.miralles@urv.cat; Tel.: +34-977-759-378

**Table S1. Biometric and biochemical data in rats after 24 weeks of dietary intervention***						
	**STD**<br>**n = 7**	**HFHS**<br>**n = 7**	**FO**<br>**n = 7**	**GSE**<br>**n = 7**	**FO + GSE**<br>**n = 7**	***p*-value†**
Body weight (g)	256.0 ± 13.9	291.6 ± 23.9[a]	318.3 ± 41.8[a]	289.8 ± 19.6[a]	291.2 ± 25.7[a]	0.005
Perigonadal WAT (g)	4.9 ± 1.1	19.6 ± 12.2[a]	19.7 ± 9.5[a]	15.5 ± 6.3[a]	12.7 ± 5.0	0.002
Liver (g)	6.7 ± 1.2	7.2 ± 0.5	6.8 ± 0.3	7.4 ± 0.7	7.7 ± 0.7	NS
Blood glucose (mmol/L)	3.58 ± 0.15	3.65 ± 0.20	3.56 ± 0.38	3.63 ± 0.26	3.72 ± 0.22	NS
Plasma insulin (mU/L)	27.27 ± 9.55	58.38 ± 15.95[a]	57.66 ± 34.23[a]	74.20 ± 33.87[a]	43.34 ± 30.10	0.008
HOMA-IR	4.38 ± 1.63	9.50 ± 2.64[a]	9.32 ± 6.08[a]	12.11 ± 6.05[a]	7.30 ± 5.20	0.012
Plasma TAG (mmol/L)	1.53 ± 0.43	1.25 ± 0.20	1.57 ±0.35	2.19 ± 0.20[a,b,c]	1.46 ± 0.27[d]	<0.001

Results were expressed as mean ± standard deviation. Abbreviations: STD, Standard; HFHS, High-Fat High-Sucrose; FO, Fish Oil; GSE, Grape Seed Extract; WAT, White Adipose Tissue; HOMA- IR, Homeostatic Assessment Model of Insulin Resistance; TAG, Triacylglycerol. *These data have already been described in previous reports [1-4]. †*p*-value (<0.05) was calculated by the non-parametric Kruskal-Wallis test followed by Mann–Whitney U test. a; vs. STD group, b; vs. HFHS group, c; vs. FO group, d; vs GSE group.

References

1. Méndez, L.; Muñoz, S.; Miralles-Pérez, B.; Rosa Nogués, M.; Ramos-Romero, S.; Torres, J.L.; Medina, I. Modulation of the liver protein carbonylome by the combined effect of marine omega-3 PUFAs and grape polyphenols supplementation in rats fed an obesogenic high fat and high sucrose diet. *Mar. Drugs* **2020**, 18, 1–30. doi:10.3390/md18010034.
2. Dasilva, G.; Pazos, M.; García-Egido, E.; Gallardo, J.M.; Ramos-Romero, S.; Torres, J.L.; Romeu, M.; Nogués, M.R.; Medina, I. A lipidomic study on the regulation of inflammation and oxidative stress targeted by marine ω-3 PUFA and polyphenols in high-fat high-sucrose diets. *J. Nutr. Biochem*. **2017**, 43, 53–67, doi:10.1016/j.jnutbio.2017.02.007.
3. Méndez, L.; Ciordia, S.; Fernández, M.S.; Juárez, S.; Ramos, A.; Pazos, M.; Gallardo, J.M.; Torres, J.L.; Nogués, M.R.; Medina, I. Changes in liver proteins of rats fed standard and high-fat and sucrose diets induced by fish omega-3 PUFAs and their combination with grape polyphenols according to quantitative proteomics. *J. Nutr. Biochem*. **2017**, 41, 84–97. doi:10.1016/j.jnutbio.2016.12.005.
4. Ramos-Romero, S.; Molinar-Toribio, E.; Pérez-Jiménez, J.; Taltavull, N.; Dasilva, G.; Romeu, M.; Medina, I.; Torres, J.L. The combined action of omega-3 polyunsaturated fatty acids and grape proanthocyanidins on a rat model of diet-induced metabolic alterations. *Food Funct*. **2016**, 7, 3516–3523. doi:10.1039/c6fo00679e.
