# Supplementary material for: Effects of Fish Oil and Grape Seed Extract Combination on Hepatic Endogenous Antioxidants and Bioactive Lipids in Diet-Induced Early Stages of Insulin Resistance in Rats
Source: Mar Drugs. 2020 Jun 16;18(6):318. doi: 10.3390/md18060318 (PMC7345288; doi:10.3390/md18060318)
Supplement: Supplementary file 1 [file marinedrugs-18-00318-s001.zip › supplementary_material/Taltavull-FishOilAndGrapeSeedExtract-TableS2.pdf]

Supplementary data:

Effects of Fish Oil and Grape Seed Extract Combination on Hepatic Endogenous Antioxidants and Bioactive Lipids in Diet-Induced Early Stages of Insulin Resistance in Rats

Núria Taltavull [1], Bernat Miralles-Pérez [1,*], Maria Rosa Nogués [1], Sara Ramos-Romero [2,3], Lucía Méndez [4], Isabel Medina [4], Josep Lluís Torres [2] and Marta Romeu [1]

[1] Universitat Rovira i Virgili, Department of Basic Medical Sciences, Pharmacology Unit, Functional Nutrition, Oxidation, and Cardiovascular Disease (NFOC-SALUT) group, C/ Sant Llorenç 21, E-43201 Reus, Spain; nuria.taltavull@urv.cat (N.T.); mariarosa.nogues@urv.cat (M.R.N.); marta.romeu@urv.cat (M.R.N.)

[2] Institute of Advanced Chemistry of Catalonia (IQAC-CSIC), C/ Jordi Girona 18-26, E-08034 Barcelona, Spain; sara.ramos@iqac.csic.es (S.R.-R.); josepllluis.torres@iqac.csic.es (J.L.T.)

[3] Department of Cell Biology, Physiology & Immunology, Faculty of Biology, University of Barcelona, E-08028 Barcelona, Spain

[4] Institute of Marine Research (IIM-CSIC), C/ Eduardo Cabello 6, E-36208 Vigo, Spain; luciamendez@iim.csic.es (L.M.); medina@iim.csic.es (I.M.)

* Correspondence: bernat.miralles@urv.cat; Tel.: +34-977-759-378

Table S2. Diacylglycerol and ceramide species identified in the rat liver									
	**Lipid specie**	**m/z***	**RT (min)**	**[M-R$_1$COOH+H]$^+$**	**[M-R$_2$COOH+H]$^+$**	**[R$_1$COO+C$_3$H$_5$O+H]$^+$**	**[R$_2$COO+C$_3$H$_5$O+H]$^+$**	**[R$_1$CO]$^+$**	**[R$_2$CO]$^+$**
SFA	16:0.16:0-DAG	586.5409	13.18	313.2737	313.2737	313.2737	313.2737	239.2375	239.2375
	16:0.16:0-DAG (II)	586.5410	13.29	313.2737	313.2737	313.2737	313.2737	239.2375	239.2375
	16:0.18:0-DAG	614.5725	13.48	341.3050	313.2737	313.2737	341.3050	239.2375	267.2688
	16:0.18:0-DAG(II)	614.5723	13.60	341.3050	313.2737	313.2737	341.3050	239.2375	267.2688
	18:0.18:0-DAG	642.6039	13.81	341.3057	341.3057	341.3050	341.3050	267.2688	267.2688
MUFA	16:0.16:1-DAG	584.5247	12.99	311.2580	313.2737	313.2737	311.2581	239.2375	237.2218
	16:0.18:1-DAG	612.5560	13.19	339.2893	313.2737	313.2737	339.2894	239.2375	265.2531
	16:0.18:1-DAG(II)	612.5567	13.28	339.2893	313.2737	313.2737	339.2894	239.2375	265.2531
	18:1.18:1-DAG	638.5720	13.27	339.2894	339.2894	339.2894	339.2894	265.2531	265.2531
PUFA	22:5.22:6-DAG	732.5551	12.30	385.2739	387.2895	387.2894	385.2737	313.2531	311.2375
	18:2.22:6-DAG	682.5406	12.45	385.2743	337.2743	337.2737	385.2737	263.2375	311.2375
	18:2.22:5-DAG	684.5557	12.55	387.2888	337.2732	337.2737	387.2894	263.2375	313.2531
	16:0.20:5+18:2.18:3-DAG	632.5247	12.50	359.2580 / 335.2580	313.2737 / 337.2737	313.2737 /337.2737	359.2581 / 335.2581	239.2375 / 263.2375	285.2218 / 261.2218
	18:1.20:5+16:0.22:6-DAG	658.5403	12.63	359.2580 / 385.2736	339.2893 / 313.2736	339.2894 / 313.2737	359.2581 / 385.2737	265.2531 / 239.2375	285.2218 / 311.2375
	18:1.22:6-DAG	684.5557	12.68	385.2732	339.2888	339.2894	385.2737	265.2531	311.2375
	16:0.22:5-DAG	660.5564	12.79	387.2893	313.2737	313.2737	387.2894	239.2375	313.2531
	18:1.22:5-DAG	686.5722	12.80	387.2895	339.2895	339.2894	387.2894	265.2531	313.2531
	16:0.18:2 + 16:0.18:2-DAG	610.5411	12.89 / 12.98	337.2737	313.2737	313.2737	337.2737	239.2375	263.2375
	18:1.18:2-DAG	636.5565	12.98	337.2741	339.2897	339.2894	337.2737	265.2531	263.2375
LCFA	Cer(d18:1/16:0)	538.5194	12.96						
	Cer(d18:1/18:1(9Z))	564.535	13.00						
	Cer(d18:1/18:0)	566.5507	13.28						
	Cer(d18:1/20:0)	594.582	13.63						
VLCFA	Cer(d18:1/24:1(15Z))	648.6289	13.94						
	Cer(d18:1/22:0)	622.6133	14.00						
	Cer(d18:1/23:0)	636.6289	14.21						
	Cer(d18:1/24:0)	650.6446	14.42						
	Cer(d18:1/25:0)	664.6602	14.60						

Abbreviations: RT, Retention time; DAG, Diacylglycerol; SFA, Saturated Fatty Acid; MUFA, Monounsaturated Fatty Acid; PUFA, Polyunsaturated Fatty Acid; LCFA, Long-Chain Fatty Acid; Cer, Ceramide; VLCFA, Very Long-Chain Fatty Acid. *DAG and ceramide species are [M+NH4]$^+$ and [M+H]$^+$, respectively.
