# Supplementary material for: Effects of Fish Oil and Grape Seed Extract Combination on Hepatic Endogenous Antioxidants and Bioactive Lipids in Diet-Induced Early Stages of Insulin Resistance in Rats
Source: Mar Drugs. 2020 Jun 16;18(6):318. doi: 10.3390/md18060318 (PMC7345288; doi:10.3390/md18060318)
Supplement: Supplementary file 1 [file marinedrugs-18-00318-s001.zip › supplementary_material/Taltavull-FishOilAndGrapeSeedExtract-TableS3.pdf]

Supplementary data:

Effects of Fish Oil and Grape Seed Extract Combination on Hepatic Endogenous Antioxidants and Bioactive Lipids in Diet-Induced Early Stages of Insulin Resistance in Rats

Núria Taltavull [1], Bernat Miralles-Pérez [1,*], Maria Rosa Nogués [1], Sara Ramos-Romero [2,3], Lucía Méndez [4], Isabel Medina [4], Josep Lluís Torres [2] and Marta Romeu [1]

[1] Universitat Rovira i Virgili, Department of Basic Medical Sciences, Pharmacology Unit, Functional Nutrition, Oxidation, and Cardiovascular Disease (NFOC-SALUT) group, C/ Sant Llorenç 21, E-43201 Reus, Spain; nuria.taltavull@urv.cat (N.T.); mariarosa.nogues@urv.cat (M.R.N.); marta.romeu@urv.cat (M.R.N.)

[2] Institute of Advanced Chemistry of Catalonia (IQAC-CSIC), C/ Jordi Girona 18-26, E-08034 Barcelona, Spain; sara.ramos@iqac.csic.es (S.R.-R.); josepIluis.torres@iqac.csic.es (J.L.T.)

[3] Department of Cell Biology, Physiology & Immunology, Faculty of Biology, University of Barcelona, E-08028 Barcelona, Spain

[4] Institute of Marine Research (IIM-CSIC), C/ Eduardo Cabello 6, E-36208 Vigo, Spain; luciamendez@iim.csic.es (L.M.); medina@iim.csic.es (I.M.)

* Correspondence: bernat.miralles@urv.cat; Tel.: +34-977-759-378

Table S3. Composition of diets					
	**STD**	**HFHS**	**FO**	**GSE**	**FO + GSE**
**Diet composition**					
Flour (g)	1000[a]	1000[b]	1000[b]	1000[b]	1000[b]
TBHQ (g)	0.08	0.08	0.08	0.08	0.08
Porcine gelatin (g)	25	25	25	25	25
Soybean lecithin (g)	6	22	22	22	22
Soybean[c] or FO[d] oil (mL)	19	24	24	24	24
GSE[f] (mg)	-	-	-	1090	1090
**Macronutrients (% weight)**					
Protein	16.4	21.7	21.7	21.7	21.7
Fat	6.2	24.1	24.1	24.1	24.1
Carbohydrates	46.6	45.0	45.0	44.9	44.9
**Macronutrients (% caloric value)**[g]					
Protein	21.3	17.9	17.9	17.9	17.9
Fat	18.2	44.9	44.9	44.9	44.9
Carbohydrates	60.5	37.2	37.2	37.2	37.2
Total energy density (kcal/g)	3.1	4.8	4.8	4.8	4.8

Abbreviations: STD, Standard; HFHS, High-Fat High-Sucrose; FO, Fish Oil; GSE, Grape Seed Extract. [a]Standard flour (Teklad Global 2014), containing wheat middlings, ground wheat, ground corn, corn gluten meal, calcium carbonate, soybean oil, dicalcium phosphate, iodized salt, l-lysine, vitamin E acetate, DL-methionine, magnesium oxide, choline chloride, manganous oxide, ferrous sulphate, menadione sodium bisulphite complex (source of vitamin K activity), zinc oxide, copper sulphate, niacin, calcium pantothenate, calcium iodate, pyridoxine hydrochloride, riboflavin, thiamin mononitrate, vitamin A acetate, vitamin B12 supplement, folic acid, cobalt carbonate, biotin and vitamin D3 supplement. [b]High-fat high-sucrose diet (Tekland TD 08811), containing sucrose (34 % of the total pellet weight), anhydrous milkfat, casein, maltodextrin, corn starch, cellulose, mineral mix AIN-93G-MX, soybean oil, vitamin mix AIN-93G-VX, L-cystine, choline, bitartrate, green food colour, tert-butylhydroquinone. [c]Soybean oil was purchased from Clearspring Ltd. (London, United Kingdom). [d]FO with eicosapentaenoic acid (EPA, C20:5 n-3) and docosahexaenoic acid (DHA, C22:6 n-3) in a balanced 1:1 ratio was obtained by mixing the appropriate quantities of the commercial fish oils AFAMPES 121 EPA (AFAMSA, Vigo, Spain), Omega-3 RX (EnerZona, Milan, Italy) and Oligen liquid DHA 80% (IFIGEN-EQUIP 98, S.L., Barcelona, Spain). [f]GSE (Fine Grajfnol®, powder 98 %) was purchased from JF-Natural Product (Tianjin, China). [g]Energy density is estimated as metabolizable energy based on the Atwater factors, assigning 4 kcal/g to protein, 9 kcal/g to fat and 4 kcal/g to carbohydrate, including dietary fibre.
