# Supplementary material for: Effects of Fish Oil and Grape Seed Extract Combination on Hepatic Endogenous Antioxidants and Bioactive Lipids in Diet-Induced Early Stages of Insulin Resistance in Rats
Source: Mar Drugs. 2020 Jun 16;18(6):318. doi: 10.3390/md18060318 (PMC7345288; doi:10.3390/md18060318)
Supplement: Supplementary file 1 [file marinedrugs-18-00318-s001.zip › supplementary_material/Taltavull-FishOilAndGrapeSeedExtract-TableS4.pdf]

Supplementary data:

Effects of Fish Oil and Grape Seed Extract Combination on Hepatic Endogenous Antioxidants and Bioactive Lipids in Diet-Induced Early Stages of Insulin Resistance in Rats

Núria Taltavull [1], Bernat Miralles-Pérez [1,*], Maria Rosa Nogués [1], Sara Ramos-Romero [2,3], Lucía Méndez [4], Isabel Medina [4], Josep Lluís Torres [2] and Marta Romeu [1]

[1] Universitat Rovira i Virgili, Department of Basic Medical Sciences, Pharmacology Unit, Functional Nutrition, Oxidation, and Cardiovascular Disease (NFOC-SALUT) group, C/ Sant Llorenç 21, E-43201 Reus, Spain; nuria.taltavull@urv.cat (N.T.); mariarosa.nogues@urv.cat (M.R.N.); marta.romeu@urv.cat (M.R.N.)

[2] Institute of Advanced Chemistry of Catalonia (IQAC-CSIC), C/ Jordi Girona 18-26, E-08034 Barcelona, Spain; sara.ramos@iqac.csic.es (S.R.-R.); josepllluis.torres@iqac.csic.es (J.L.T.)

[3] Department of Cell Biology, Physiology & Immunology, Faculty of Biology, University of Barcelona, E-08028 Barcelona, Spain

[4] Institute of Marine Research (IIM-CSIC), C/ Eduardo Cabello 6, E-36208 Vigo, Spain; luciamendez@iim.csic.es (L.M.); medina@iim.csic.es (I.M.)

[*] Correspondence: bernat.miralles@urv.cat; Tel.: +34-977-759-378

Table S4. Fatty acid composition of diets

	STD	HFHS and GSE	FO and FO + GSE
14:00	0.017	11.5	11.6
16:00	14.8	32.2	32.0
16:1 n-7	0.015	1.7	1.7
18:00	2.9	13.7	13.7
18:1n-9	20.5	26.4	26.2
18:1 n-7	0.026	0.67	0.68
18:2 n-6	58.6	9.7	8.7
20:00	n.d.	n.d.	0.0069
18:3 n-3	2.9	1.1	1.1
20:1 n-9	0.025	0.031	0.021
18:4 n-3	0.0026	0.0032	0.033
20:2 n-6	0.0034	0.0043	0.0045
20:3 n-6	n.d.	n.d.	0.0048
20:4 n-6	0.0069	0.0086	0.036
22:1 n-11	0.019	0.023	0.024
22:1 n-9	0.0043	0.0054	0.006
20:4 n-3	0.0034	0.0043	0.022
20:5 n-3	0.012	0.015	0.54
24:1 n-9	0.0048	0.0060	0.0082
22:5 n-3	0.0045	0.0056	0.093
22:6 n-3	0.02	0.024	0.56
Total SFAs	17.5	59.4	60.5
Total MUFAs	21.1	29.3	28.4
Total PUFAs	61.1	10.8	10.7
Total n-3	3.3	1.6	1.4
Total n-6	58.8	10.4	10.6

Results are expressed as a percentage of total fatty acids (mg/100 mg of total fatty acids). Abbreviations: STD, Standard; HFHS, High-fat High-sucrose; FO, Fish Oil; GSE, Grape Seed Extract; SFA, Saturated Fatty Acid; MUFA, Monounsaturated Fatty Acid; PUFA, Polyunsaturated Fatty Acid; n.d., non-detected.
