# Supplementary material for: Effects of Fish Oil and Grape Seed Extract Combination on Hepatic Endogenous Antioxidants and Bioactive Lipids in Diet-Induced Early Stages of Insulin Resistance in Rats
Source: Mar Drugs. 2020 Jun 16;18(6):318. doi: 10.3390/md18060318 (PMC7345288; doi:10.3390/md18060318)
Supplement: Supplementary file 1 [file marinedrugs-18-00318-s001.zip › supplementary_material/Taltavull-FishOilAndGrapeSeedExtract-TableS5.pdf]

# Supplementary data:

# Effects of Fish Oil and Grape Seed Extract Combination on Hepatic Endogenous Antioxidants and Bioactive Lipids in Diet-Induced Early Stages of Insulin Resistance in Rats

**Núria Taltavull [1], Bernat Miralles-Pérez [1,*], Maria Rosa Nogués [1], Sara Ramos-Romero [2,3], Lucía Méndez [4], Isabel Medina [4], Josep Lluís Torres [2] and Marta Romeu [1]**

[1] Universitat Rovira i Virgili, Department of Basic Medical Sciences, Pharmacology Unit, Functional Nutrition, Oxidation, and Cardiovascular Disease (NFOC-SALUT) group, C/ Sant Llorenç 21, E-43201 Reus, Spain; nuria.taltavull@urv.cat (N.T.); mariarosa.nogues@urv.cat (M.R.N.); marta.romeu@urv.cat (M.R.N.)

[2] Institute of Advanced Chemistry of Catalonia (IQAC-CSIC), C/ Jordi Girona 18-26, E-08034 Barcelona, Spain; sara.ramos@iqac.csic.es (S.R.-R.); joseplluis.torres@iqac.csic.es (J.L.T.)

[3] Department of Cell Biology, Physiology & Immunology, Faculty of Biology, University of Barcelona, E-08028 Barcelona, Spain

[4] Institute of Marine Research (IIM-CSIC), C/ Eduardo Cabello 6, E-36208 Vigo, Spain; luciamendez@iim.csic.es (L.M.); medina@iim.csic.es (I.M.)

* Correspondence: bernat.miralles@urv.cat; Tel.: +34-977-759-378

| Table S5. Characterization used for the liver histological study | |
|---|---|
| **Meaning Liver** | **Grade** |
| **Steatosis** | |
| Physiological state | < 5% |
| Low-grade | 5-33% |
| Middle-grade | 33-66% |
| High-grade | > 66% |
| **Localization of steatosis** | |
| Centrilobular | 0 |
| Periportal | 1 |
| No zonal | 2 |
| **Lobular inflammation** | |
| No | 0 |
| < 2 focus | 1 |
| 2-4 focus | 2 |
| > 4 focus | 3 |
| **Portal inflammation** | |
| No | 0 |
| Yes | 1 |
